# Transcriptional Consequences of MeCP2 Knockdown and Overexpression in Mouse Primary Cortical Neurons

**DOI:** 10.3390/ijms26189032

**Published:** 2025-09-17

**Authors:** Mostafa Rezapour, Joshua Bowser, Christine Richardson, Metin Nafi Gurcan

**Affiliations:** 1 Wake Forest Institute for Regenerative Medicine (WFIRM), Wake Forest University School of Medicine, Winston-Salem, NC 27101, USA; 2 Department of Biological Sciences, University of North Carolina at Charlotte, Charlotte, NC 28223, USAc.richardson@charlotte.edu (C.R.); 3 Center for Artificial Intelligence Research, Wake Forest University School of Medicine, Winston-Salem, NC 27101, USA

**Keywords:** MeCP2, Rett syndrome, autism spectrum disorder, neurodevelopmental disorder, differential expression analysis, RNA-seq, biomarker discovery, GLMQL-MAS, Cross-MAS, mouse cortical neurons

## Abstract

Rett syndrome (RTT) and MECP2 duplication syndrome, a subtype of autism spectrum disorder (ASD), are neurodevelopmental disorders caused by MeCP2 loss and gain of function, respectively. While MeCP2 is known to regulate transcription through its interaction with methylated DNA and chromatin-associated factors such as topoisomerase IIβ (TOP2β), the downstream transcriptional consequences of MeCP2 dosage imbalance remain partially characterized. Here, we present a transcriptome-centered analysis of mouse primary cortical neurons subjected to MeCP2 knockdown (KD) or overexpression (OE), which model RTT and ASD-like conditions in parallel. Using a robust computational pipeline integrating generalized linear models with quasi-likelihood F-tests and Magnitude–Altitude Scoring (GLMQL-MAS), we identified differentially expressed genes (DEGs) in KD and OE relative to wild-type (WT) neurons. This study represents a computational analysis of secondary transcriptomic data aimed at nominating candidate genes for future experimental validation. Gene Ontology enrichment revealed both shared and condition-specific biological processes, with KD uniquely affecting neurodevelopmental and stress-response pathways, and OE perturbing extracellular matrix, calcium signaling, and neuroinflammatory processes. To prioritize robust and disease-relevant targets, we applied Cross-MAS and further filtered DEGs by correlation with MeCP2 expression and regulation directional consistency. This yielded 16 high-confidence dosage-sensitive genes that were capable of classifying WT, KD, and OE samples with 100% accuracy using PCA and logistic regression. Among these, RTT-associated candidates such as Plcb1, Gpr161, Mknk2, Rgcc, and Abhd6 were linked to disrupted synaptic signaling and neurogenesis, while ASD-associated genes, including Aim2, Mcm6, Pcdhb9, and Cbs, implicated neuroinflammation and metabolic stress. These findings establish a compact and mechanistically informative set of MeCP2-responsive genes, which enhance our understanding of transcriptional dysregulation in RTT and ASD and nominate molecular markers for future functional validation and therapeutic exploration.

## 1. Introduction

Rett syndrome (RTT) is a severe neurodevelopmental disorder predominantly caused by mutations in the X-linked Methyl-CpG binding protein 2 (MeCP2) gene [1]. However, loss-of-function mutations in MeCP2 are not limited to RTT and can also give rise to a broader group of MeCP2-related disorders (MeCP2-pathies), which include intellectual disability (ID) and ASD-like clinical presentations [2]. MeCP2 is a methyl-cytosine binding protein that modulates gene expression by binding to methylated CA (mCA) and CG (mCG) dinucleotides within gene bodies, particularly in long neuronal genes [3]. This binding is thought to repress transcription by impeding RNA polymerase II elongation, with the extent of repression proportional to the density of methylated sites [3]. Acting primarily as a transcriptional repressor, MeCP2 silences or reduces gene expression by binding to methylated DNA [4]. It also represses the activity of topoisomerase IIβ (TOP2β) at regulatory regions of long genes, thus influencing how easily these genes are transcribed [4].

TOP2β is a type II topoisomerase that facilitates transcription by resolving DNA supercoiling and inducing transient double-stranded breaks (DSBs), particularly in long genes [5]. In neurons, where TOP2β is highly expressed, this function is essential for the expression of genes involved in synaptic signaling, plasticity, and other neurodevelopmental processes [6,7]. Disruption of MeCP2 leads to aberrant TOP2β activity, which results in abnormal expression of long genes and contributes to RTT-associated pathophysiology [4].

Although MeCP2 has traditionally been viewed as a transcriptional repressor, findings indicate that it also modulates gene expression in more complex and context-dependent ways [8,9]. MeCP2 binds broadly across the genome, and its effects on transcription are modest and context-dependent [3,4,10]. Moreover, MeCP2 levels are high in mature neurons and reach a one-to-one ratio with nucleosomes, reflecting its global chromatin-associated regulatory role [2,11]. Subtle but widespread transcriptional changes observed in MeCP2-deficient and overexpressing models highlight the importance of tightly regulated MeCP2 dosage in maintaining neuronal homeostasis [3,12].

While MeCP2 deficiency causes RTT, MeCP2 overexpression leads to MECP2 duplication syndrome, a condition characterized by autism spectrum disorder (ASD)-like features, intellectual disability, and motor abnormalities [13,14]. These opposing phenotypes underscore the dosage sensitivity of MeCP2 in neurons and emphasize the need to study both loss and gain of function within a unified framework.

Recent work by Nettles et al. [4] demonstrated that MeCP2 physically interacts with TOP2β and modulates its activity at promoters and enhancers of long neuronal genes. Using eTIP-seq and ChIP-seq, they showed that MeCP2 loss or overexpression alters TOP2β activity. While Nettles et al. [4] provided valuable mechanistic insight into how MeCP2 modulates TOP2β activity, their work focused primarily on biochemical interactions and profiling of enzyme activity at the genome-wide level in neurons.

In contrast to the study by Nettles et al. [4], the current study adopts a transcriptome-centered approach to investigate how perturbations in MeCP2 dosage, specifically knockdown (KD) and overexpression (OE), alter gene expression patterns in neurons. While KD models the MeCP2 loss of function observed in RTT [15,16], OE has been implicated in autism spectrum disorder (ASD) [17]. Thus, analyzing both perturbations in parallel enables a systematic comparison of RTT- and ASD-related transcriptional mechanisms driven by MeCP2 imbalance.

The central aim of this study is to identify protein-coding genes that are transcriptionally associated with MeCP2 and exhibit dosage-sensitive expression in opposite directions, downregulated in KD and upregulated in OE, making them strong candidates for capturing the distinct molecular signatures underlying RTT and ASD. We hypothesize that a distinct subset of genes will respond to MeCP2 dosage in a directionally consistent and disease-relevant manner.

To this end, differentially expressed genes (DEGs) under KD and OE conditions, relative to the wild type (WT), are identified using the generalized linear model with quasi-likelihood F-tests and Magnitude–Altitude Score (GLMQL-MAS) [18,19,20,21,22], which establishes the foundational transcriptional changes associated with MeCP2 dosage shifts. Subsequent Gene Ontology (GO) enrichment analysis [23] of significantly regulated DEGs, defined by an absolute log2 fold change greater than one and a Benjamini–Hochberg adjusted *p*-value [24,25] below 0.05, reveals distinct biological processes uniquely enriched in each condition, highlighting the asymmetric and non-reciprocal effects of MeCP2 loss and gain.

To isolate genes most specifically associated with each perturbation, Cross-MAS [21] is employed to exclude DEGs common to both KD and OE, thus allowing for the identification of uniquely responsive protein-coding genes based on effect size and statistical significance, with MeCP2 gene explicitly excluded. This refinement ensures that the analysis emphasizes genes whose expression is selectively affected by MeCP2 dosage rather than general transcriptional stress or compensatory responses.

Further prioritization is achieved by filtering for genes that exhibit strong Spearman correlation [26] with MeCP2 gene expression and demonstrate directionally consistent regulation, upregulated in OE or downregulated in KD, which enriches for candidates that are likely to represent direct or indirect MeCP2 targets. The potential of these genes to serve as molecular signatures of MeCP2 status is evaluated using principal component analysis (PCA) [27] and logistic regression [28,29], which assess their capacity to classify WT, KD, and OE samples and support their relevance for transcriptomic profiling and potential diagnostic applications.

Finally, functional characterization using g:Profiler [30] determines whether these dosage-sensitive genes are enriched in biological functions and pathways linked to RTT, which enhances the interpretability of the results and nominates new candidates for further investigation.

While the mechanistic effects of MeCP2 dosage have been explored, a critical gap remains in identifying transcriptional markers that could serve as molecular surrogates for MeCP2 dysfunction in clinical settings. Collectively, this study establishes an integrative transcriptomic framework to identify MeCP2-associated, dosage-sensitive genes that are statistically robust, biologically relevant, and potentially translational. By jointly analyzing MeCP2 KD and OE conditions, representing RTT and ASD phenotypes, respectively, the study advances current understanding of how MeCP2 dosage imbalances alter neuronal gene expression and nominates condition-specific markers for RTT and ASD. In this study, we aim not only to elucidate the transcriptomic consequences of MeCP2 imbalance, but also to identify candidate genes that could inform diagnostic or therapeutic strategies in RTT and MECP2 duplication syndrome.

## 2. Results

To investigate the impact of MeCP2 dosage alterations on gene expression, we compared transcriptomes from KD and OE neuronal conditions against WT controls using GLMQL-MAS [18,19,20,21,22]. Differentially expressed genes (DEGs) were identified using thresholds of |log2 fold change| > 1 (the notation “|.|” denotes the absolute value) and Benjamini–Hochberg (BH)-adjusted *p*-value < 0.05 [24,25].

**Figure 1:**
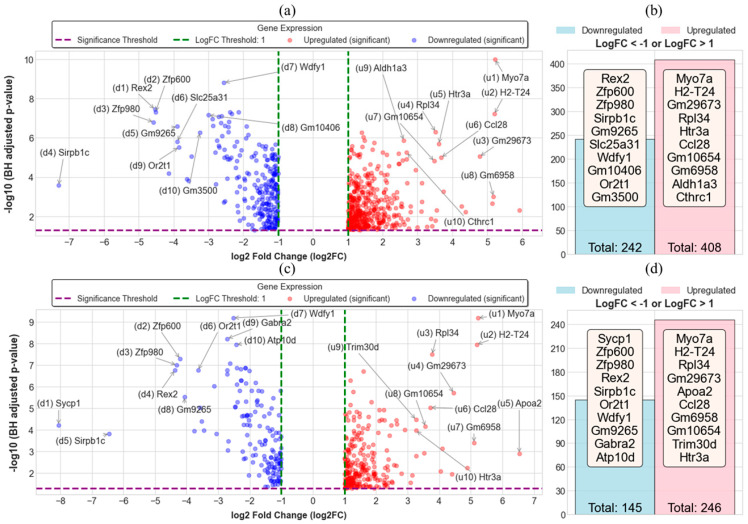
Transcriptomic changes in neurons following MeCP2 knockdown (KD) and overexpression (OE) relative to wild type (WT). (**a**) Volcano plot of significant differentially expressed genes (DEGs) in KD vs. WT, highlighting the top 10 genes ranked by GLMQL-MAS, with u1–u10 denoting the top-ranked upregulated genes and d1–d10 representing the top-ranked downregulated genes. (**b**) Summary of DEG counts showing significantly upregulated (logFC > 1) and downregulated (logFC < −1) genes in KD vs. WT. (**c**) Volcano plot of DEGs in OE vs. WT with top 10 ranked genes labeled. (**d**) DEG counts in OE vs. WT comparison. All DEGs were identified using BH-adjusted *p*-value < 0.05 and |log2 fold change| > 1 thresholds.

Figure 1a presents the DEGs identified in the KD vs. WT comparison. Only statistically significant genes are shown, with the top 10 most strongly affected genes labeled based on GLMQL-MAS ranking. In this comparison, 242 genes were significantly downregulated, and 408 were significantly upregulated, as summarized in Figure 1b. Similarly, the OE vs. WT contrast is shown in Figure 1c, where the most prominent DEGs are annotated. The distribution of significant DEGs includes 145 downregulated and 246 upregulated genes (Figure 1d).

**Figure 2:**
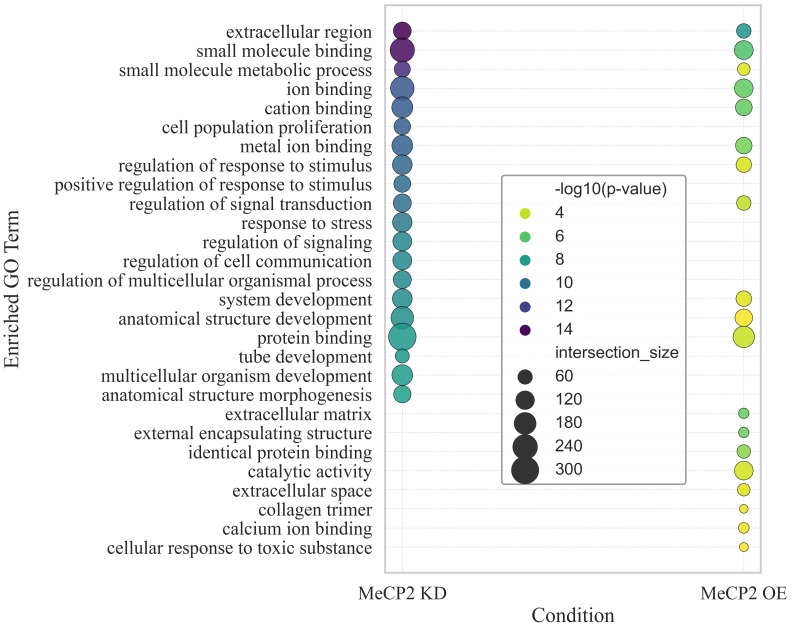
GO enrichment analysis of significant genes from MeCP2 KD and OE conditions. Each dot represents a GO term enriched among the top 20 most significant terms per condition, sized by intersection size (number of associated genes) and colored by −log10(*p*) value. Common and unique GO terms are visualized between KD and OE, revealing shared processes such as anatomical structure development and ion binding, as well as condition-specific functions such as tube development (KD) and extracellular matrix organization (OE).

To gain insight into the biological functions perturbed by MeCP2 dosage alterations, we performed GO enrichment analysis on all significantly differentially expressed genes from both KD and OE conditions. Using the top 20 enriched GO terms (ranked by −log10(*p*) value) per condition, we identified overlapping and unique functional categories associated with each state. As shown in Figure 2, multiple GO terms were shared between KD and OE, thus suggesting common pathways affected by both loss and gain of MeCP2 function. These include broad categories such as anatomical structure development, extracellular region, cation binding, protein binding, and system development, among others.

In contrast, distinct sets of GO terms were uniquely enriched in each condition. For MeCP2 KD, uniquely enriched terms included cell population proliferation, tube development, response to stress, and regulation of multicellular organismal processes, thus indicating disruption of growth and communication pathways. For MeCP2 OE, the unique enrichment profile involved terms such as extracellular matrix, collagen trimer, calcium ion binding, and cellular response to toxic substances, thus implicating processes linked to structural remodeling and stress response.

To delineate the gene expression changes that are uniquely associated with either MeCP2 KD or OE, as well as those common to both conditions, we applied the Cross-MAS algorithm [21]. This analysis also focused exclusively on statistically significant DEGs (BH-adjusted *p* < 0.05) with |log2 fold change| > 1.

**Figure 3:**
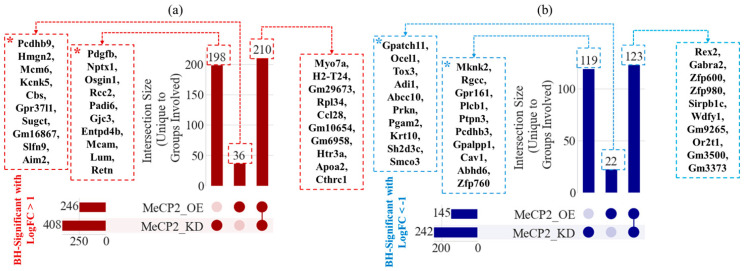
Identification of unique and shared differentially expressed genes (DEGs) in MeCP2 KD and OE conditions using Cross-MAS. (**a**) UpSet plot showing intersections of significantly upregulated genes (logFC > 1, BH-adjusted *p* < 0.05) across KD and OE relative to WT. Top 10 genes from each group are annotated. (**b**) UpSet plot of significantly downregulated genes (logFC < −1, BH-adjusted *p* < 0.05) with the top 10 genes for each unique or shared set labeled. Note: The gene MeCP2 was removed from the lists to avoid redundancy and focus on downstream targets. Genes marked with an asterisk (*) indicate those selected for downstream analyses, as they were uniquely and significantly up- or downregulated in either KD or OE, but not both.

Figure 3a presents the intersection analysis for significantly upregulated genes. Among these, 198 genes were upregulated exclusively in KD, 36 in OE, and 210 genes were upregulated in both KD and OE. The most strongly upregulated genes unique to KD included Pdgfb, Nptx1, Osgin1, and Rcc2, whereas OE-specific upregulation featured genes such as Pcdhb9, Hmgn2, Mcm6, and Kcnk5. Genes commonly upregulated in both conditions included Myo7a, H2-T24, Rpl34, and Apoa2, reflecting a core transcriptional response to altered MeCP2 dosage.

Figure 3b shows the equivalent analysis for significantly downregulated genes. Here, 119 genes were downregulated only in KD, 22 only in OE, and 123 were shared between both conditions. KD-specific downregulation included Mknk2, Rgcc, Gpr161, and Plcb1, while OE-specific genes included Gpatch11, Ocel1, and Tox3. Shared downregulated genes comprised the top two genes Rex2 and Gabra2. Note that the top downregulated gene in KD was MeCP2 itself; however, it was excluded from the reported list to focus on downstream targets.

**Figure 4:**
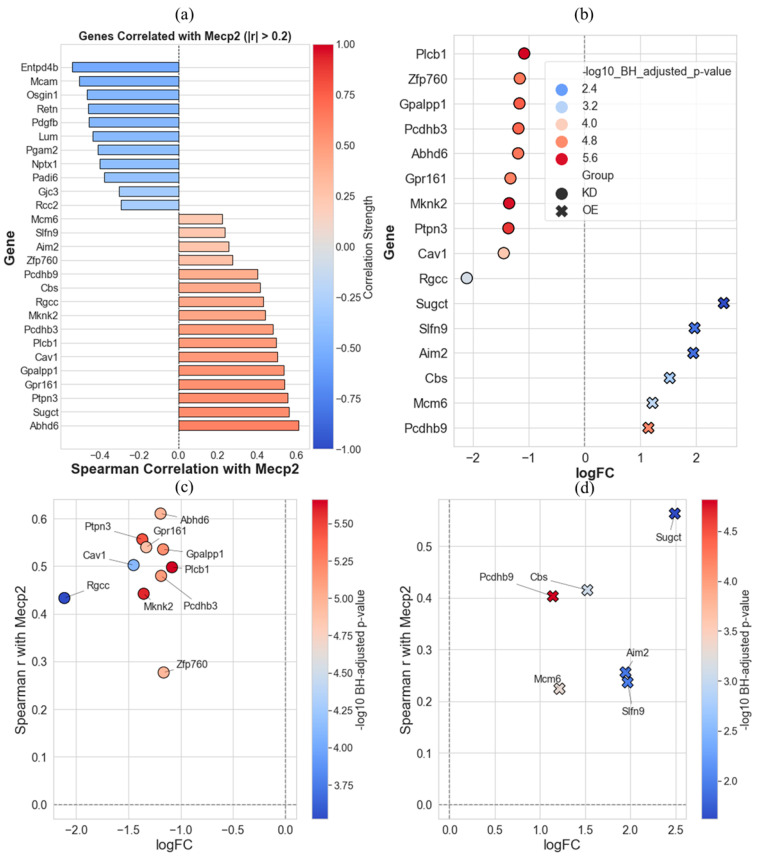
Prioritization of dosage-sensitive MeCP2-associated genes based on correlation and expression directionality. (**a**) Spearman correlation coefficients between MeCP2 expression and 40 candidate genes selected from Figure 3, filtered to retain those with absolute correlation > 0.2. (**b**) Subset of genes from panel (**a**) that were significantly downregulated in KD (logFC < 0) or upregulated in OE (logFC > 0), visualized by logFC and grouped by condition. Dot color reflects −log10 BH-adjusted *p*-value. All retained genes showed expression changes consistent with MeCP2 correlation direction. (**c**) Scatterplot of logFC versus Spearman correlation in KD, which highlights directionally consistent and significantly downregulated genes. (**d**) Same as (**c**) but for OE, which shows significantly upregulated genes. Top genes are labeled.

To further refine the list of MeCP2-associated genes, we conducted correlation-based prioritization of candidates previously identified as uniquely up- or downregulated in MeCP2 KD or OE conditions (asterisk-labeled in Figure 3). Figure 4a shows the Spearman correlation coefficients between MeCP2 expression and the expression levels of 40 selected genes, filtered to include only those with absolute correlation values greater than 0.2. Figure 4b then narrows this list further by retaining only genes that were downregulated in KD (logFC < 0) or upregulated in OE (logFC > 0). This filtering step captures genes that are transcriptionally responsive to MeCP2 dosage shifts in a directionally consistent manner, i.e., reduced expression when MeCP2 is lost, or elevated expression when MeCP2 is overexpressed.

Note that since our goal was to identify representative signatures for KD and OE, we needed to define directionality with respect to MeCP2 levels. While the opposite pattern (genes upregulated in KD and downregulated in OE) could also reflect dosage sensitivity, we focused on this direction of regulation to isolate genes whose expression positively tracks with MeCP2 dosage, thus enhancing interpretability and biological coherence.

To visualize how these dosage-sensitive genes relate to MeCP2 expression more precisely, we plotted logFC against Spearman correlation values separately for KD (Figure 4c) and OE (Figure 4d). These panels illustrate the subset of genes that not only respond to MeCP2 dosage perturbations but also exhibit coherent expression dynamics with MeCP2 itself. For instance, in KD (Figure 4c), genes like Abhd6 and Ptpn3 show strong downregulation and positive correlation with MeCP2, thus indicating likely repression due to MeCP2 loss. Conversely, in OE (Figure 4d), genes like Sugct and Pcdhb9 are both upregulated and positively correlated, thus reinforcing their role as putative MeCP2-responsive targets.

To evaluate the discriminatory power of the selected genes, we applied dimensionality reduction using PCA followed by classification using logistic regression. These genes were identified through an integrative pipeline involving GLMQL-MAS, Cross-MAS, correlation analysis, and logFC-directional agreement. The final set of selected genes includes Pcdhb9, Gpr161, Slfn9, Gpalpp1, Mcm6, Zfp760, Abhd6, Pcdhb3, Plcb1, Cav1, Rgcc, Mknk2, Cbs, Sugct, Aim2, and Ptpn3.

**Figure 5:**
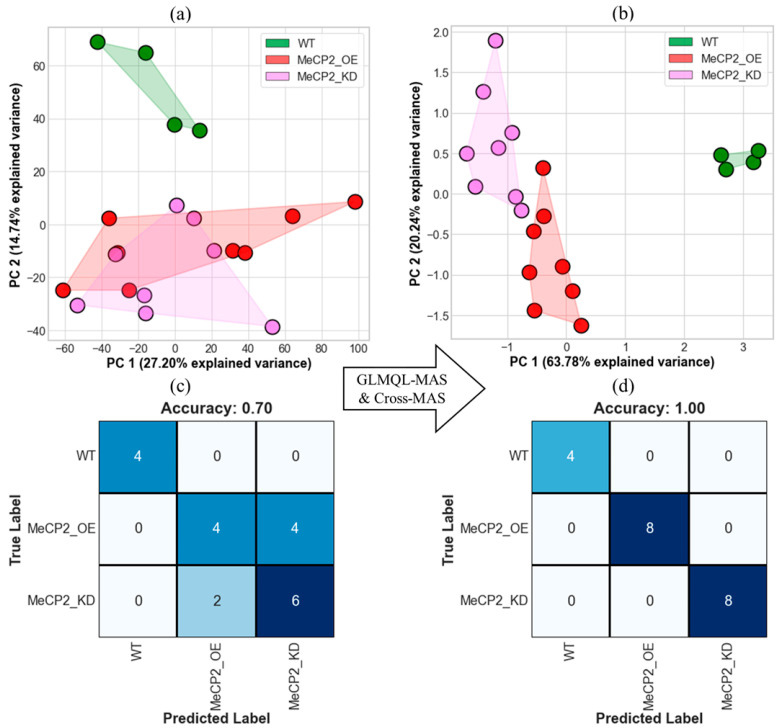
Discriminative power of Cross-MAS-selected genes for classifying MeCP2 dosage states. (**a**) PCA plot using all 21,202 protein-coding genes shows partial overlap among WT, MeCP2_KD, and MeCP2_OE groups. (**b**) PCA using only the 16 selected genes from Figure 4c results in complete group separation. (**c**) Confusion matrix showing 70% accuracy from logistic regression using PC1 and PC2 of all protein-coding genes as predictors. (**d**) Confusion matrix showing 100% accuracy when using PC1 and PC2 of the 16 selected genes.

Figure 5a displays the PCA of all 21,202 protein-coding genes, where sample clusters overlap between KD and OE conditions. In contrast, PCA based on the 16 selected genes (Figure 5b) yields complete separation between the three groups, with no overlap among the WT, KD, and OE clusters. This qualitative improvement is quantitatively supported by the performance of logistic regression models trained on the first two principal components (PC1 and PC2). Principal components were used as predictors rather than raw gene expression values to reduce model dimensionality and prevent overfitting, particularly given the relatively small sample size.

Using all protein-coding genes (Figure 5c), classification accuracy reached 70%, with misclassifications primarily between KD and OE. When using only the 16 selected genes (Figure 5d), classification accuracy improved to 100%. These results demonstrate that the selected DEGs capture the core transcriptional differences induced by altered MeCP2 dosage and serve as effective features for group classification. The ability of these 16 genes to distinguish MeCP2 dosage states with 100% classification accuracy suggests the potential for developing diagnostic panels. These could support patient stratification or monitoring of therapeutic response in clinical contexts where MeCP2 expression is dysregulated.

**Figure 6:**
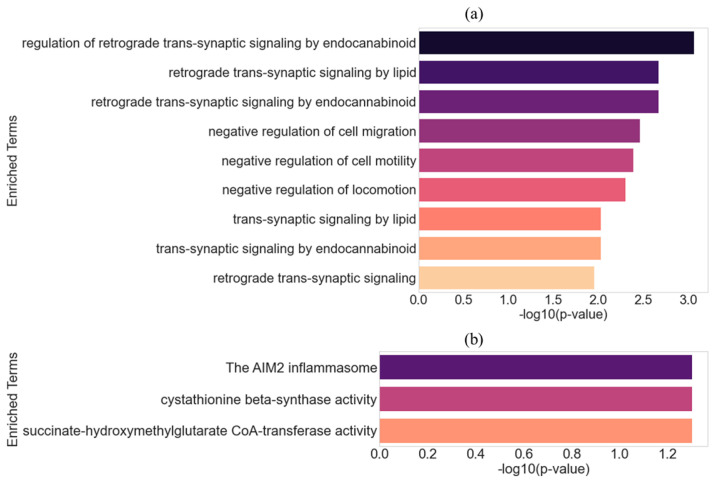
Functional enrichment of dosage-sensitive MeCP2-associated genes. Bar plot showing the top enriched BP terms identified using g:Profiler based on the 16 genes selected through GLMQL-MAS, Cross-MAS, correlation analysis, and logFC agreement. (**a**) Enriched biological processes from the selected 10 genes downregulated in MeCP2 knockdown (KD), including terms related to retrograde trans-synaptic signaling, cell migration, and lipid signaling, processes commonly disrupted in RTT. (**b**) Enriched terms from the selected 6 genes upregulated in MeCP2 overexpression (OE), highlighting pathways linked to neuroinflammation and metabolic stress, including the AIM2 inflammasome and CBS activity, which are relevant to ASD-related pathology.

Using the 16 selected genes, we next performed functional enrichment analysis to determine whether these dosage-sensitive genes are involved in biological processes relevant to RTT. Enrichment was conducted using g:Profiler [30] with the Mus musculus background. The analysis identified several significantly enriched biological process (BP) terms (Figure 6). Figure 6a illustrates enrichment results for the KD-specific genes, i.e., Cav1, Abhd6, Ptpn3, Gpalpp1, Plcb1, Gpr161, Pcdhb3, Mknk2, Zfp760, and Rgcc. The top terms include regulation of retrograde trans-synaptic signaling by endocannabinoid, negative regulation of cell migration, and trans-synaptic signaling by lipid. Figure 6b shows the enrichment profile for the OE-specific genes, i.e., Sugct, Aim2, Slfn9, Cbs, Pcdhb9, and Mcm6. The top terms include the AIM2 inflammasome, cystathionine beta-synthase activity, and succinate-hydroxymethylglutarate CoA-transferase activity.

## 3. Discussion

The robust transcriptional changes observed following MeCP2 knockdown and overexpression (Figure 1) underscore the dosage-sensitive role of MeCP2 in neuronal gene regulation. The asymmetric distribution of DEGs, with a higher number of upregulated genes in both KD and OE conditions, suggests that MeCP2 may function predominantly as a repressor in neurons. Notably, several genes, such as Myo7a, H2-T24, and Gm29673, were significantly upregulated in both knockdown and overexpression conditions, thus suggesting the existence of a core transcriptional program that is consistently activated by any disruption to MeCP2 dosage. This bidirectional sensitivity implies that even opposing changes in MeCP2 levels can converge on shared molecular pathways, which may reflect compensatory mechanisms or common stress responses that arise from chromatin dysregulation.

Our GO enrichment analysis (Figure 2) revealed that MeCP2 dosage alterations impact a broad spectrum of biological processes, with both shared and condition-specific effects that reflect the molecular complexity of disorders associated with MeCP2 imbalance, including RTT and ASD. Notably, MeCP2 KD, which models RTT [16], uniquely enriched GO categories such as cell population proliferation, multicellular organism development, tube development, and response to stress. These terms align with the developmental arrest, impaired neurogenesis, and elevated cellular stress responses that characterize RTT pathology [31].

It is important to note, however, that MeCP2 loss of function is not restricted to RTT; it is also implicated in a wider spectrum of MeCP2-related disorders (MeCP2-pathies), which include intellectual disability and ASD-like features [2]. Therefore, the transcriptional changes observed in mouse cortical neurons in this study should be interpreted within the broader framework of MeCP2-pathies rather than being limited to RTT-specific pathology.

MeCP2 OE, which has been implicated in ASD-like phenotypes, including in MECP2 duplication syndrome [17], showed unique enrichment in terms such as calcium ion binding, extracellular matrix, collagen trimer, and cellular response to toxic substance. Disrupted calcium signaling is a well-established feature in ASD, with mutations in calcium channel genes such as Cacna1c and Cacna1h contributing to abnormal synaptic activity and neuronal excitability [32,33]. Likewise, altered responses to toxic substances and increased oxidative stress have been reported in ASD, thus suggesting impairments in detoxification and stress response pathways [34,35]. These distinctions underscore that MeCP2 dosage changes produce non-redundant transcriptional outcomes, with KD primarily disrupting developmental regulation, and OE affecting extracellular structure and neurotoxic stress signaling.

The intersection and correlation analyses (Figure 3 and Figure 4) identified 16 genes that exhibited directionally consistent expression changes and significant correlation with MeCP2 levels. Several of these genes converge on pathways linked to neuronal signaling, synaptic remodeling, and cellular stress, hallmark processes known to be disrupted in RTT.

Pdgfb encodes platelet-derived growth factor subunit B (PDGFB), a key growth factor involved in processes such as cell proliferation, angiogenesis, and tissue repair [36]. Overexpression of Pdgfb has been consistently associated with vascular abnormalities and multiple cancers [37]. The functional PDGFBB dimer has been shown to protect primary hippocampal neurons from glutamate-induced injury via PDGFR-β-signaling activation [38]. In the context of RTT, upregulation of Pdgfb may indicate aberrant neurovascular signaling or compensatory mechanisms attempting to mitigate neuronal stress and damage.

Nptx1 encodes neuronal pentraxin 1 (NP1), which plays a key role in synaptic remodeling and activity-dependent plasticity [39,40]. As a neuron-specific gene, Nptx1 may be sensitive to chromatin changes and transcriptional misregulation in MeCP2-deficient states [12]. While increased NP1 can enhance synaptic refinement, excessive levels have been implicated in triggering apoptosis [41], suggesting that Nptx1 upregulation in RTT models may contribute to synaptic instability and neurodegeneration.

Osgin1 (oxidative stress-induced growth inhibitor 1) is involved in the regulation of autophagy, apoptosis, and ferroptosis in response to oxidative stress [42]. Oxidative stress is an increasingly recognized factor in RTT-related neuronal dysfunction [31]. Elevated expression of Osgin1 may reflect heightened cellular stress and could exacerbate neuronal loss through apoptotic pathways [43,44], further contributing to the neurodegenerative features of RTT.

Mknk2 encodes MAP kinase-interacting serine/threonine-protein kinase 2 (MNK2), which plays a regulatory role in mRNA translation by phosphorylating eukaryotic initiation factor 4E (eIF4E) [45]. This modulation of translation initiation is important for controlling cellular responses to stress and growth signals [46,47]. Downregulation of Mknk2 can impair eIF4E phosphorylation, leading to translational repression and disrupted protein synthesis [48], a mechanism increasingly implicated in neurodevelopmental disorders such as RTT [49].

Rgcc is involved in controlling progression through the G1/S and G2/M phases of the cell cycle [50]. Reduced expression of Rgcc can lead to failures in cell cycle checkpoint control, resulting in abnormal proliferation or apoptosis [51]. Since precise regulation of cell proliferation is important during brain development, Rgcc downregulation under MeCP2 deficiency could contribute to the impaired neurogenesis and structural deficits observed in RTT.

Gpr161 encodes a G protein-coupled receptor that modulates the Hedgehog signaling pathway, which is important for proper neural tube patterning and central nervous system development [52]. Downregulation of Gpr161 can lead to hyperactivation of Hedgehog signaling, which disrupts developmental programs and causes structural abnormalities [52]. Alterations in this pathway have been implicated in neurodevelopmental disorders, including autism spectrum disorder, which shares clinical features with RTT [53,54]. Thus, reduced Gpr161 expression in MeCP2 KD neurons may contribute to overlapping phenotypic features.

Plcb1 encodes phospholipase C beta 1 (PLCB1), a key enzyme in phosphoinositide signaling that regulates intracellular calcium levels, neuronal excitability, and synaptic plasticity [55]. Reduced Plcb1 expression impairs downstream signaling from metabotropic glutamate receptor 5 (mGluR5), which affects calcium dynamics and gene expression important for neuronal communication and development [56]. Downregulation of Plcb1 has been associated with synaptic dysfunction, seizures, and motor impairments—hallmark features of RTT pathology [57].

In contrast to the RTT-associated genes described above, several genes identified in the MeCP2 OE condition are linked to ASD-related mechanisms. AIM2 is part of an inflammasome complex that activates caspase-1 and induces pro-inflammatory cytokines like IL-1β and IL-18. Studies in children with ASD demonstrate elevated AIM2 inflammasome activation and increased IL-1β/IL-18 levels compared to controls [58]. Elevated AIM2 signaling correlates with neuroimmune dysregulation observed in ASD [59]. Recent genomic analyses have identified de novo missense MCM6 variants in individuals with ASD, developmental delay, and epilepsy [60]. The Simons Foundation Autism Research Initiative (SFARI) also classifies MCM6 as a strong candidate ASD gene (Category 2) due to recurring damaging mutations in ASD cohorts [61].

These findings confirm that the transcriptional response to MeCP2 imbalance involves genes with functions directly tied to synaptic regulation, calcium signaling, oxidative stress response, and neurodevelopmental patterning. By specifically analyzing downregulated genes in the knockdown condition, we identified candidate molecular signatures linked to RTT-related mechanisms. Conversely, the upregulated genes under MeCP2 overexpression highlight pathways associated with ASD, including neuroinflammation and structural remodeling. The consistent dosage-sensitive behavior of these genes, combined with their correlation to MeCP2 levels and their established roles in neuronal development and disease, positions them as promising molecular markers for MeCP2-driven dysfunction and potential targets for future mechanistic and therapeutic investigations in both RTT and ASD contexts.

To evaluate whether the selected genes could function as effective classifiers of MeCP2 dosage states, we applied PCA, followed by logistic regression, using the 16 prioritized genes identified through GLMQL-MAS, Cross-MAS, correlation analysis, and directionality filtering. As shown in Figure 5a, PCA using the entire transcriptome (21,202 protein-coding genes) resulted in partial overlap between KD and OE clusters, thus indicating substantial transcriptional heterogeneity and limiting discriminatory power. In contrast, PCA using only the 16 selected genes (Figure 5b) achieved complete separation of WT, KD, and OE samples, clearly delineating the three dosage conditions. This qualitative improvement was supported by quantitative classification performance: logistic regression using the full transcriptome reached 70% accuracy, with most errors being between KD and OE (Figure 5c), while models trained on the 16 selected genes achieved perfect classification (100% accuracy, Figure 5d). These results underscore the ability of these genes to capture core transcriptional differences that are most reflective of MeCP2 dosage changes. Their compact and non-redundant profile makes them highly suitable for distinguishing perturbed MeCP2 states within this in vitro dataset and suggest potential utility in transcriptome-based signatures for RTT and ASD classification. However, these findings should be interpreted with caution, as they are derived from a single in vitro dataset of cultured mouse cortical neurons and require further validation in vivo before translational applications can be considered.

To further characterize the biological relevance of these 16 genes, we performed separate functional enrichment analyses for those downregulated in KD and upregulated in OE (Figure 6). The KD-specific genes were enriched in processes such as retrograde trans-synaptic signaling, endocannabinoid signaling, and regulation of cell migration, which are tightly linked to synaptic connectivity and neural plasticity [62], core domains disrupted in RTT. In contrast, the OE-specific genes were enriched in pathways related to the AIM2 inflammasome, CBS enzymatic activity, and mitochondrial metabolism, thus highlighting neuroinflammatory and metabolic processes implicated in ASD [58,63,64]. These findings reinforce the functional distinction between MeCP2 loss and gain, and provide mechanistic support for the relevance of the identified genes to RTT and ASD pathophysiology.

## 4. Materials and Methods

### 4.1. Dataset Description

The RNA-seq data analyzed in this study were obtained from Nettles et al. [4], available via the Gene Expression Omnibus (GEO) under accession number GSE246463. In their experimental setup, primary cortical neurons were cultured from embryonic day 14.5 (E14.5) C57BL/6J mouse embryos. Dissociated cortical cells were plated on poly-D-lysine-coated plates and maintained in neurobasal medium supplemented with B27, GlutaMAX, fetal bovine serum, and antibiotics.

Neurons were cultured in vitro for 12 days (DIV12), with lentiviral transduction performed on DIV3 to induce either MeCP2 knockdown (KD) or MeCP2 overexpression (OE). Control samples were transduced with a non-targeting virus. Each experimental group included both technical and biological replicates, with neuronal dissection and transduction performed independently across days for biological replication.

Total RNA was extracted using the RNeasy Mini Kit (Qiagen) and subjected to rRNA depletion. RNA libraries were constructed using the NEBNext Ultra Directional RNA Library Prep Kit and sequenced on an Illumina NextSeq 500 platform, which produced single-end 75 bp reads with typical yields of 20–40 million reads per sample.

This RNA-seq dataset was generated as part of a broader investigation into MeCP2 interactions with topoisomerase IIβ (TOP2β). However, for the present study, only the transcriptomic data were used to assess gene expression changes under MeCP2 KD and OE conditions relative to wild-type (WT) neurons.

### 4.2. Statistical Methods

To identify differentially expressed genes (DEGs) under altered MeCP2 dosage conditions, we employed the GLMQL-MAS framework [18,19,20,21,22], which integrates generalized linear models (GLMs) [65] with quasi-likelihood (QL) F-tests [66] and Magnitude–Altitude Scoring (MAS) [67,68]. This approach is well-suited for RNA-seq count data due to its ability to model the inherent overdispersion typically observed in transcriptomic datasets, where the variance often exceeds the mean [69].

RNA-seq count data were first normalized using the Trimmed Mean of M-values (TMM) normalization [70] method to adjust for library size differences and compositional bias across samples. After normalization, dispersion estimates were computed to model biological variability. A design matrix was constructed with the wild type (WT) as the reference group, and two separate contrasts were defined: one comparing MeCP2 knockdown (KD) vs. WT, and the other comparing MeCP2 overexpression (OE) vs. WT. For each contrast, a Quasi-Likelihood (QL) F-test was performed using a full model that included the respective MeCP2 condition (KD or OE), and a reduced model that excluded it. This modeling approach allows for robust differential expression analysis tailored to each perturbation, while accounting for the overdispersed nature of RNA-seq data.

Genes were defined as significantly differentially expressed if they met both of the following criteria: (1) BH-adjusted *p*-value < 0.05 (Benjamini–Hochberg method for multiple testing correction [24,25]); and (2)|log2 fold change| > 1, that is, logFC > 1 or logFC < −1. To enhance biological interpretability and prioritize genes showing both statistical and biological relevance, we computed a Magnitude–Altitude Score (MAS) for each gene using the following formula:

(1) $${MAS}_{l}={|{log}_{2}(\mathrm{FC}}_{l}{)|}^{M}|{{log}_{10}(p}_{l}^{BH}){|}^{A},$$

where ${\mathrm{FC}}_{l}$ is the fold change and $p_{l}^{BH}$ is the BH-adjusted *p*-value for gene l, and both exponents M and A were set to 1 to give equal weight to expression magnitude and statistical confidence.

To systematically distinguish between condition-specific and shared transcriptional changes, we further applied Cross-MAS, a ranking-based algorithm designed to identify genes with consistently strong MAS scores across multiple comparisons [21]. For each gene, Cross-MAS identifies its highest MAS-based rank across all condition pairs and selects those genes with the lowest maximum ranks. This min–max optimization strategy effectively pinpoints genes that demonstrate robust and reproducible expression changes in KD, OE, or both. In this ranking system, rank 1 corresponds to the gene with the largest MAS score (Equation (1)), thus denoting both a large |logFC| and small adjusted *p*-value.

To refine the high-confidence DEGs identified by GLMQL-MAS and Cross-MAS, we applied an additional prioritization step based on correlation with MeCP2 expression and directionality of regulation. Specifically, we calculated Spearman correlation coefficients [26] between MeCP2 and each DEG across all samples to capture monotonic relationships between gene expression and MeCP2 levels, which may reflect dosage-sensitive transcriptional control. Only genes exhibiting an absolute Spearman correlation coefficient greater than 0.2 were retained for further analysis, thereby focusing on those with at least moderate association to MeCP2 expression.

To further enrich for biologically consistent and potentially direct MeCP2-responsive genes, we filtered for directionality agreement: genes that were downregulated in KD (logFC < 0) and upregulated in OE (logFC > 0). This criterion ensures that selected genes display expression patterns coherent with MeCP2 dosage, decreasing when MeCP2 is depleted and increasing when it is overexpressed. By intersecting correlation-based filtering with directionality agreement, we identified a compact set of dosage-sensitive genes whose transcriptional responses not only met statistical thresholds and ranking criteria but also aligned with the expected MeCP2-driven regulatory behavior. These genes were subsequently evaluated for their discriminative power and biological relevance using principal component analysis, classification modeling, and functional enrichment.

Specifically, principal component analysis (PCA) [27] was performed using expression values transformed as the logarithm base 2, and the first two principal components (PC1 and PC2) were used as input features in a logistic regression model. This analysis was applied to the same dataset to evaluate how well these selected genes could discriminate among WT, KD, and OE conditions, rather than to build a predictive model.

### 4.3. Gene Ontology (GO) Analysis

To interpret the functional implications of the DEGs and selected genes, we performed Gene Ontology (GO) analysis using the g:Profiler web server [30] through its Python client GProfiler (version v1.0.0, accessed via the gprofiler-official package).

For each condition, gene symbols from the sets of significantly upregulated and downregulated genes were extracted and submitted as input queries to GProfiler, specifying the mouse genome (organism = “mmusculus”). Enrichment was performed against the GO database, including the biological process, cellular component, and molecular function categories. Only GO terms identified as statistically significant according to g:Profiler’s internal multiple testing correction procedure were retained for analysis. All enrichment procedures were conducted at the gene level, using GProfiler’s default statistical model and annotation framework, without applying a custom background gene set.

## 5. Conclusions

This study presents a comprehensive transcriptome-centered investigation of MeCP2 dosage imbalance in neurons by analyzing both knockdown (KD) and overexpression (OE) conditions relative to wild type (WT). Using an integrative computational framework combining GLMQL-MAS, Cross-MAS, correlation analysis, and directionality filtering, we identified a compact set of 16 protein-coding genes that exhibit robust, directionally consistent, and MeCP2-correlated expression changes. These genes effectively distinguish KD, OE, and WT states with 100% classification accuracy in our dataset, thus highlighting their potential as dosage-sensitive transcriptional signatures. Future in vivo validation will be necessary to confirm their robustness and generalizability.

Functional enrichment analysis revealed that genes downregulated in KD, modeling MeCP2 loss of function and RTT, were significantly enriched in biological processes related to synaptic connectivity, neuronal development, and retrograde trans-synaptic signaling. Among these, Plcb1, Gpr161, Mknk2, Rgcc, Ptpn3, Cav1, and Abhd6 emerged as key RTT-associated candidates. These genes are linked to disrupted calcium signaling, cell cycle regulation, and impaired synaptic plasticity, features consistent with RTT pathophysiology.

In contrast, OE-specific genes, associated with MeCP2 gain of function and ASD, were enriched in pathways related to neuroinflammation, extracellular matrix remodeling, and metabolic stress. Notable ASD-related candidates include Aim2, Mcm6, Pcdhb9, Slfn9, Sugct, and Cbs, which implicate inflammasome activation, mitochondrial dysfunction, and structural signaling abnormalities, hallmarks of MECP2 duplication syndrome and broader ASD pathology.

Together, these findings demonstrate that MeCP2 loss and gain produce non-redundant transcriptional outcomes that reflect distinct disease etiologies. The identified gene set provides mechanistic insight into the differential molecular consequences of MeCP2 dosage perturbation and nominates candidates for future functional studies. This work represents a computational analysis of secondary transcriptomic data, designed to prioritize candidate genes for experimental follow-up and translational exploration. While these results are encouraging, they should be viewed as preliminary until validated in vivo, and their translational potential for diagnostic marker development or therapeutic targeting in RTT, ASD, and related MeCP2-pathies will require further confirmation.

### Limitations of Study and Future Work

Although this study presents a robust transcriptome-centered framework for identifying MeCP2 dosage-sensitive genes, two limitations should be acknowledged. First, the analysis was based entirely on a single in vitro RNA-seq dataset derived from cultured mouse cortical neurons, which may not fully capture the complexity and cellular diversity of the in vivo brain environment. This constraint could limit the generalizability of the findings to broader neurodevelopmental contexts. We recognize that experimental validation, particularly in in vivo models or additional in vitro systems, would significantly strengthen the biological interpretation of our findings. However, the present study is designed as a hypothesis-generating investigation based on transcriptomic data and not as a validation study. Future research will aim to pursue these validations through targeted follow-up experiments.

Second, to the best of our knowledge, there are no other publicly available datasets that provide MeCP2 knockdown and overexpression transcriptomes under matched experimental conditions, thus preventing direct validation of the identified gene signatures in independent systems.

Future work should aim to replicate these findings using in vivo models and include diverse brain regions and developmental stages, which would better capture the full spectrum of MeCP2 function.

## Data Availability

The RNA-seq dataset analyzed in this study was originally published by Nettles et al. [4] and is publicly available through the Gene Expression Omnibus (GEO) under accession number GSE246463. Only the transcriptomic portion of the dataset was used in this study to investigate gene expression changes in MeCP2 knockdown (KD) and overexpression (OE) relative to wild-type (WT) neurons.
